# Hippocampal-dependent navigation in head-fixed mice using a floating real-world environment

**DOI:** 10.1038/s41598-024-64807-w

**Published:** 2024-06-21

**Authors:** Sarah A. Stuart, Jon Palacios-Filardo, Aleks Domanski, Matt Udakis, Ian Duguid, Matt W. Jones, Jack R. Mellor

**Affiliations:** 1 Centre for Synaptic Plasticity, School of Physiology, Pharmacology and Neuroscience, University of Bristol, Bristol, BS8 1TD UK; 2 Centre for Discovery Brain Sciences, Edinburgh Medical School: Biomedical Sciences, University of Edinburgh, Edinburgh, EH8 9XD UK

**Keywords:** Neuroscience, Cellular neuroscience, Cognitive neuroscience, Learning and memory, Synaptic plasticity

## Abstract

Head-fixation of mice enables high-resolution monitoring of neuronal activity coupled with precise control of environmental stimuli. Virtual reality can be used to emulate the visual experience of movement during head fixation, but a low inertia floating real-world environment (mobile homecage, MHC) has the potential to engage more sensory modalities and provide a richer experimental environment for complex behavioral tasks. However, it is not known whether mice react to this adapted environment in a similar manner to real environments, or whether the MHC can be used to implement validated, maze-based behavioral tasks. Here, we show that hippocampal place cell representations are intact in the MHC and that the system allows relatively long (20 min) whole-cell patch clamp recordings from dorsal CA1 pyramidal neurons, revealing sub-threshold membrane potential dynamics. Furthermore, mice learn the location of a liquid reward within an adapted T-maze guided by 2-dimensional spatial navigation cues and relearn the location when spatial contingencies are reversed. Bilateral infusions of scopolamine show that this learning is hippocampus-dependent and requires intact cholinergic signalling. Therefore, we characterize the MHC system as an experimental tool to study sub-threshold membrane potential dynamics that underpin complex navigation behaviors.

## Introduction

Understanding how neural activity leads to complex behaviors is a central goal of neuroscience. Activity in individual neurons, dendrites and synapses determines behavioral outcomes but measurement and manipulation of activity in such small compartments during behavior is challenging, particularly in freely moving animals. The widespread adoption of head-fixed recording platforms enables physical stability for high-resolution measurements^1–4^ but with an associated trade-off: cognitive abilities are reduced due to restricted animal movement and sensory input, and increased stress levels, although the latter may be ameliorated by appropriate habituation^5–10^.

The hippocampus encodes context-dependent memories using information from multiple sensory modalities^6,11–13^. In rodents, hippocampal-dependent memory processing is most commonly measured by the encoding of spatial location, where mice or rats are asked to remember specific locations in a 2 dimensional (or sometimes 3 dimensional) environment^11,14–16^. Often this is coupled with measurement of place cell activity, which represents the neural correlate of spatial location and provides a unique coupling between behavior and neural activity^17,18^. Place cell activity may be measured using implanted electrodes or miniaturised microscopes in freely moving mice or rats but for membrane potential or sub-cellular resolution measurements whole-cell patch clamp or 2-photon microscopy is often employed requiring enhanced physical stability.

For spatial navigation tasks commonly used in rodents, head-fixation is frequently combined with virtual reality where mice are trained to run on a treadmill or floating ball to navigate virtual environments^3,4^. This confers many benefits including readily adaptable environments, but it also requires rodents to navigate almost exclusively by visual and proprioceptive cues and has largely precluded navigation in 2 dimensions^7,19^, with a few notable exceptions^9,10,20^. The lack of 2D movement limits the development of complex spatial navigation tasks in a head-fixed system. One solution to this problem is to use a floating real-world environment that moves around the animal when it moves. Such a system requires an arena that is not anchored to the recording setup and moves freely in response to animal movement^21^.

The Mobile Homecage (MHC) system allows 2D movement in an air-lifted (floating) real-world environment, with potential benefits for animal welfare^22–24^. However, the MHC itself may have limitations—freely moving animals use both near and far visual cues to navigate their environment—but in the MHC animals must principally rely on near visual (and somatosensory) cues since the animal’s position relative to far external cues does not change as they explore.

We therefore designed an experiment in our adapted MHC to test whether mice are able to perform a spatial learning task that relies solely on local maze cues and examine the flexibility of this learned behaviour when reward contingencies are shifted. We first assessed the encoding of environment in hippocampus by measuring place cell activity and demonstrated feasibility of stable whole-cell patch clamp recording in awake running mice. We then assessed dependence of spatial reversal learning in the MHC on intact cholinergic signalling in the dorsal hippocampus.

## Results

**Figure 1:**
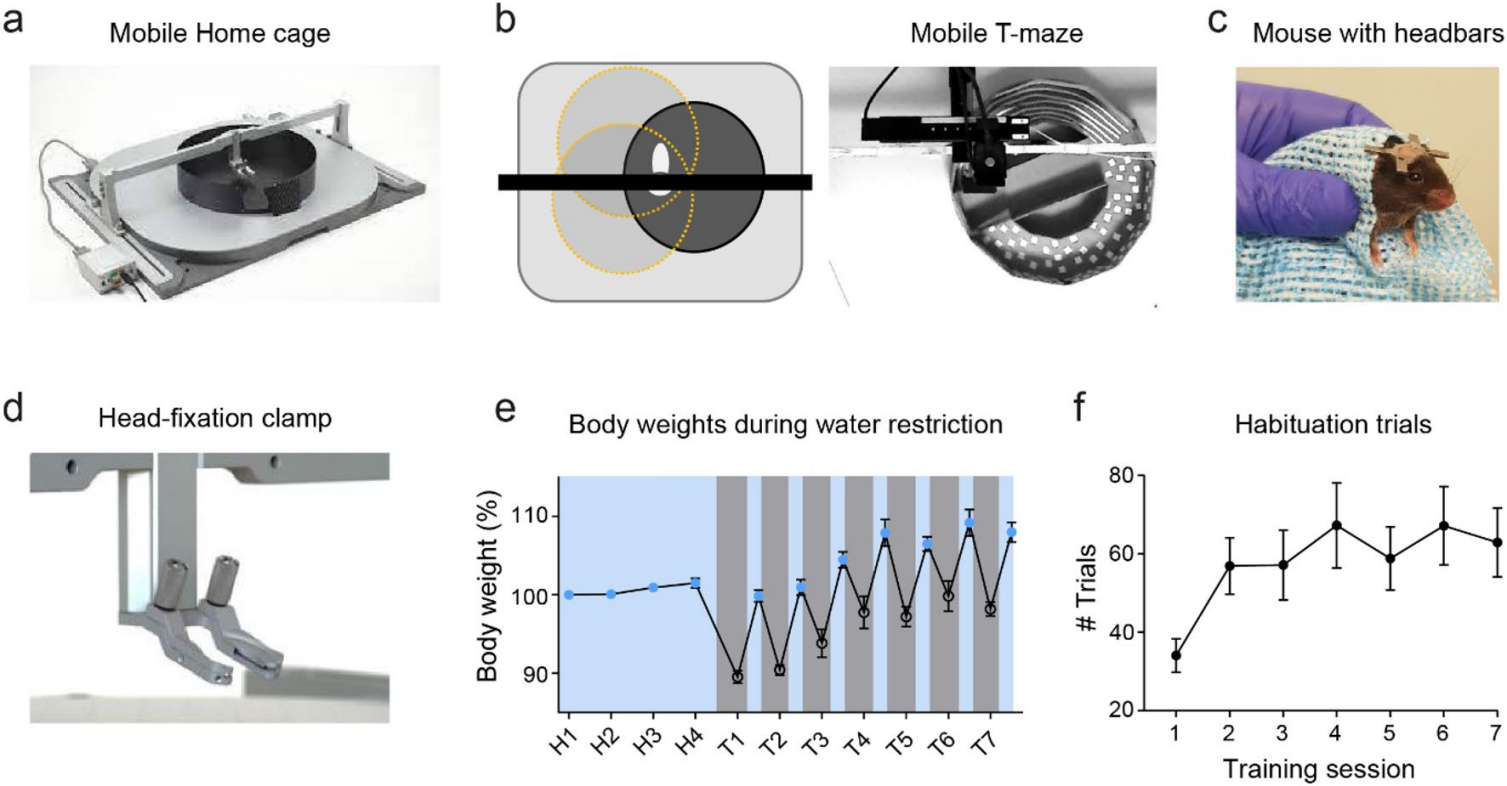
Overview of the Mobile Homecage system. (**a**) Head-fixed apparatus: the air-lifted carbon fibre container (seen in black, without foam maze insert) placed under the head-fixation bridge on the air-dispensing table. (**b**) Diagramatic view of the head-fixed apparatus (left): the mouse is able to move the container while remaining fixed in place at the bridge. Real-world view showing the container with foam T maze insert (right). (**c**) Mouse wrapped in flannel for transfer between the home cage and the head-fixed apparatus. (**d**) Head-fixation clamp angled at 37°. (**e**) Effect of water restriction on body weight over consecutive days. Blue indicates periods of ad libitum water and grey indicates absence of water. (**f**) Number of completed trials (laps of circular track) in 15 min sessions across consecutive days (n = 12 mice). Data shown as mean ± SEM.

We first developed a standardised method for habituating mice to head-fixation and the MHC apparatus (Fig. 1a,b), based on prior work^23^. All animals underwent head-plate attachment surgery followed by 4 days of post-operative care. Five days after the surgery each mouse was handled by the experimenter using a tunnel and cup handling method^25^ until they readily exited the tunnel into the experimenter’s palm. Animals were then habituated to a cloth-wrapping procedure used to transfer the animals into the head-fixed apparatus (Fig. 1c). The mice were placed in the head-fixed apparatus for 15 min with the floating container underneath. A modified, angled clamp was used to mimic the natural head position of a freely moving animal and allow engagement of the whiskers with both walls and floor (Fig. 1d). In addition, a visor was fixed over the eyes to limit visual cues from above and external to the MHC walls. To enhance motivation to explore the arena, animals underwent an overnight water restriction protocol (Fig. 1e), commencing from the night prior to the first head-fixation session. Animals received a liquid reward (~ 4 μl of 10% sucrose) delivered via a lick port when they completed a full lap of the circular track. We observed the activity of an initial cohort of mice in 15 min sessions across 7 consecutive days to assess habituation to the head-fixation over time. The number of completed laps increased after the first session of head-fixation and stabilised across the remaining test days (Fig. 1f).

### Hippocampal CA1 place cell activity in the MHC in the absence of external maze cues

Mice have been shown to rely on extra-maze cues to determine their relative location within an environment, but it has been suggested that when these cues become unreliable or absent, animals become more dependent on local cues for spatial navigation^6,7,22,24^. In the MHC, the mice are head-fixed such that their position relative to external maze cues does not change as they move the air-lifted environment around them, and external maze cues are largely obscured by the visor. Therefore, the animals are likely to rely on the visual and tactile cues within the maze, as well as egocentric cues, in order to learn the position of an otherwise unmarked reward location.

We first tested hippocampal representations of the MHC arena with an annular insert restricting mice to running around the edge of the arena and assessed whether the available local sensory cues are sufficient to endow CA1 place cell firing patterns with spatial information similar to that found in freely moving recordings or mice behaving in virtual reality.

**Figure 2:**
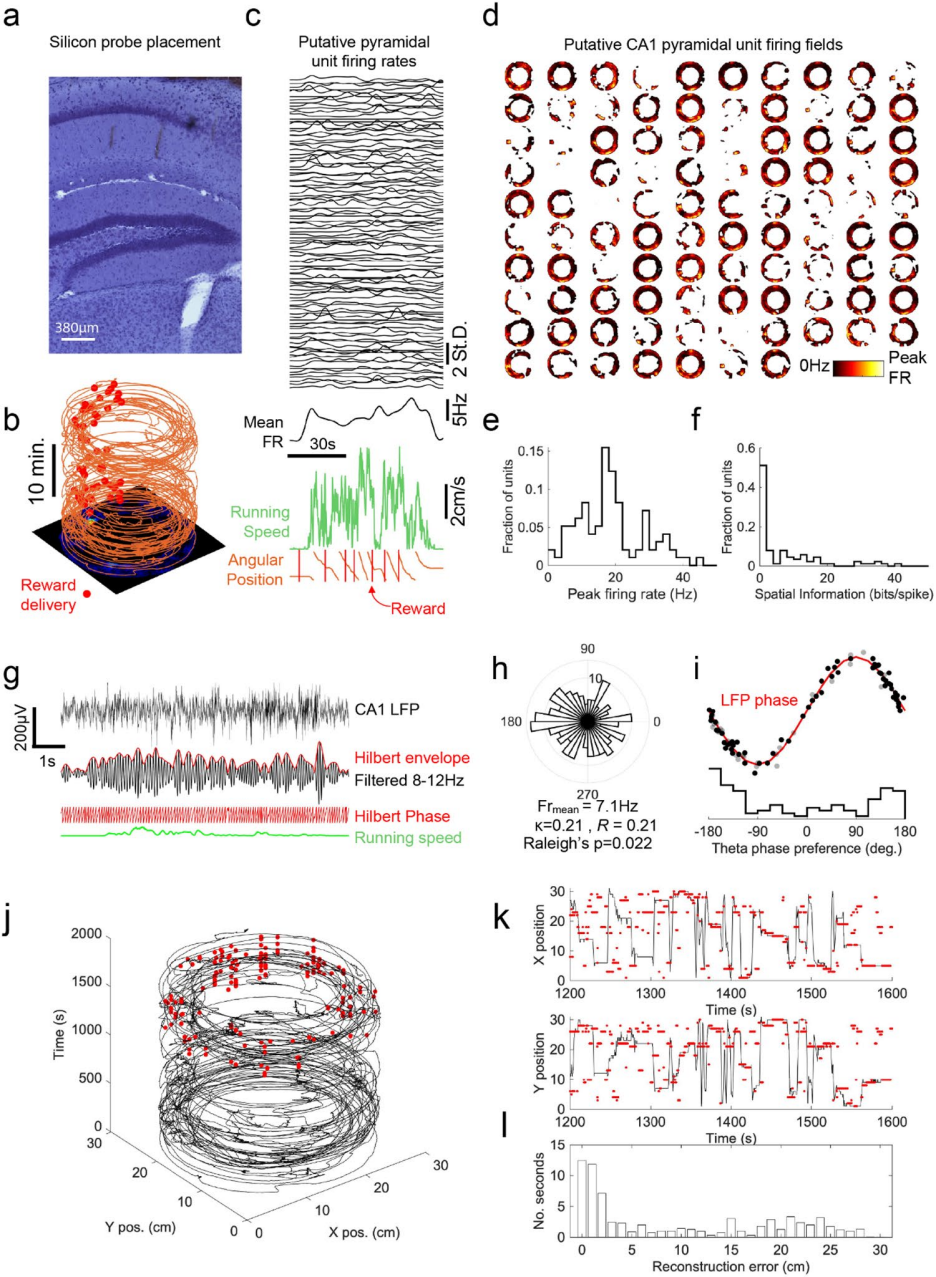
Hippocampal CA1 place cell activity during circular track running in the MHC. (**a**) Post-mortem electrolytic lesions spanning recording locations in dorsal hippocampus. (**b**) Positional tracking data showing repeated laps around the annular track, with reward locations marked by red dots (left panel). (**c**) Time-resolved normalized firing rates (top panel) aligned to 80 s of running speed data encompassing 7 laps of the track (bottom panel). (**d**) averaged spatial firing rate maps of 97 simultaneously recorded CA1 putative pyramidal cells. Distributions of peak firing rates (**e**) and spatial information content (**f**) across the population. (**g**) 8.5 s snapshot of wideband (upper trace) and 8–12 Hz bandpass filtered (middle traces) LFP from a recording site in the CA1 pyramidal cell layer, exemplifying variation of theta rhythm amplitude and phase (red traces) with running speed (lower trace, green). (**h**) Phase-locking histogram of an example place cell, showing significantly non-uniform distribution of spike times relative to theta phase (circular statistics values noted alongside phase histogram). (**i**) Theta phase preference across the population of 97 putative pyramidal cells, showing averaged preferred firing phase clustered around the downward phase of the theta cycle (red line); preferred phase of significantly phase-locked units (Rayleigh p < 0.05) shown with black dots, non-significantly locked units in grey. (**j**) Actual positional tracking data (black trace) with positions decoded from spike times marked as red dots for the second 15 min of the session; Bayesian decoder trained on spikes and locations from first 15 min. (**k**) Example of 400 s of positional decoding, with tracked x and y positions shown by black line and positions estimated by the Bayesian decoder in red dots. (**l**) Histogram showing distribution of positional error estimates across decoded time bins, showing low error during majority of bins.

To measure place cell activity, a silicon probe with 4 shanks each housing 16 contacts was acutely inserted into the dorsal CA1 region of the hippocampus whilst a mouse ran laps of the arena over a period of 30 min (Fig. 2a,b). A reward was delivered once per lap at the same location. 97 putative CA1 pyramidal cell units were identified that were active during running, showing distributions of firing rates and spatial information (Fig. 2c–f) commensurate with similar experiments in freely moving or virtual reality environments^6,26,27^. A prominent theta rhythm was evident in the local field potential (LFP) from the silicon probe and putative CA1 pyramidal cell firing was strongly entrained to the theta cycle as previously described (Fig. 2g–i). To test the ability for the spatial information recorded in CA1 to predict mouse location on the circular track, we trained a Bayesian decoder on the entire population of unit activity during the first 15 min of the recording session and then tested the predicted location against the actual location during the second 15 min of the recording session (Fig. 2j–l). For most of the session the decoder was able to accurately predict location indicating that place cell ensembles formed accurate representations of the MHC environment.

**Figure 3:**
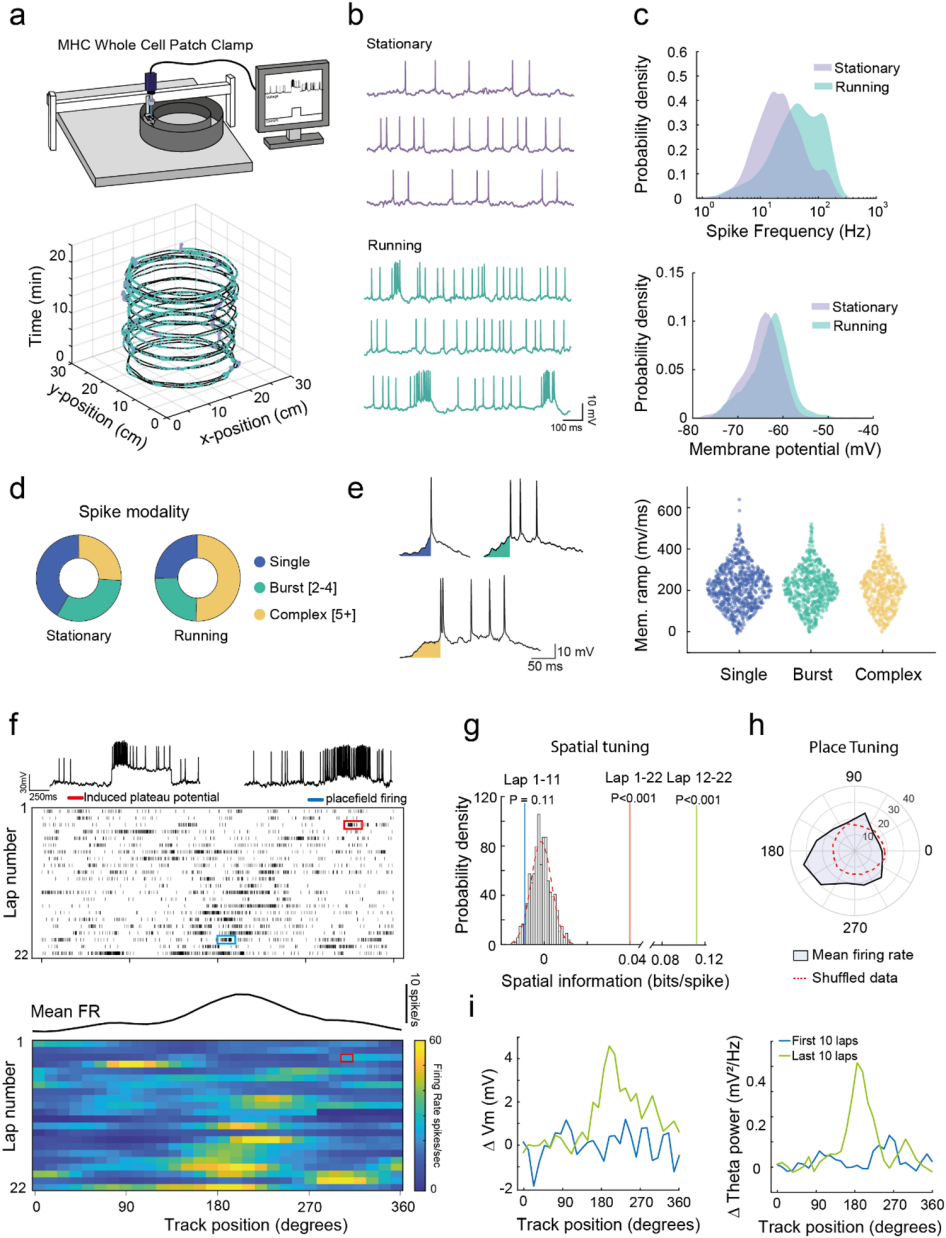
Patch clamp recording from hippocampal CA1 pyramidal neurons enables induction of place field tuning. (**a**) Depiction of whole-cell patch clamp recording of mice in MHC (top). Position coordinates of mouse running in circular MHC environment with overlayed spike times during running (green) and stationary (purple) epochs (bottom). (**b**) Example intracellular voltage recordings during running and stationary epochs. (**c**) Probability density distribution of spike frequency (top), and underlying membrane potential (bottom) for both running and stationary epochs. (**d**) Spike modality proportions for running and stationary epochs. (**e**) Example traces of single, burst and complex spikes with preceding ‘ramp’ depolarisation highlighted (left). Distribution of ramp depolarisation integrals for the three spike modalities across both running and stationary epochs (right). (**f**) Spike time raster plot for recording shown in A. Induction of plateau potential induced during lap three and resulting place field firing in lap 20 (top). Mean firing rate across all laps and heatmap of average firing rate binned across the length of the track, demonstrating development of place field firing after plateau potential induction (bottom). (**g**) Spatial information for the entire, first half and last half of the recording compared to the spatial information of shuffled data demonstrating development of spatial encoding after plateau potential induction. (**h**) Place cell tuning preference across entire recording compared to shuffled data. (**i**) Underlying membrane voltage (left) and membrane theta power (right) as mouse traverses across place field for first and last half of the recording.

Head-fixed configurations are ideal for performing whole-cell patch clamp recordings during behavior due to enhanced physical stability. Whole-cell recordings are the optimal method to measure underlying sub-threshold membrane potential dynamics, gain understanding of the synaptic inputs to individual neurons during behavior and can also be used to induce synaptic plasticity and place fields in specific pyramidal cells^1,3,28–30^. To assess the physical stability of the MHC apparatus we made whole-cell patch clamp recordings from putative CA1 pyramidal neurons whilst mice ran in the circular track (Fig. 3a,b). CA1 pyramidal cells were identified by depth of recording electrode and characteristic electrophysiological responses to current injections. We obtained 13 recordings of cells from 7 mice lasting > 1 min with an average recording duration of 279 ± 79 s (interquartile range 114–390 s). The average resting membrane potential and input resistance was − 62.9 ± 1.2 mV and 109 ± 9 MΩ respectively and the average action potential frequency and amplitude was 10.3 ± 2.2 Hz and 30 ± 3 mV respectively. In the example shown, membrane potential was depolarised and the spike frequency was elevated during running in comparison to stationary with cells often firing in bursts of action potentials, as expected for CA1 pyramidal cells (Fig. 3c,d). These results demonstrate feasibility of whole-cell recording and the physical stability of the MHC system.

Whole-cell recording also enabled analysis of sub-threshold membrane potential dynamics. It is proposed that different rates of membrane potential depolarization give rise to different spike patterns, i.e. single vs bursts of action potentials^29,31^. However, we found that membrane potential ramp gradients driving action potentials were not different between single spikes and bursts (Fig. 3e).

It has also been shown that spontaneously occurring long duration (> 50 ms) depolarisations termed plateau potentials can induce location specific firing in CA1 pyramidal cells via Behavioral Timing Synaptic Plasticity (BTSP)^29,30,32,33^. In this scenario place cell firing evolves at locations experienced just prior to the plateau potential due to the asymmetric nature of BTSP. Plateau potentials can also be induced artificially, mimicking membrane depolarization by current injection through the patch pipette^29,30,32^. When we applied current injection for 750 ms to induce an artificial plateau potential the firing pattern of the pyramidal cell developed spatial tuning in subsequent laps preferentially firing in an area of the track immediately prior to the location of the plateau potential (Fig. 3f–i). This demonstrates the potential of the mobile homecage as an environment to test plasticity of spatial representations.

### Flexible spatial reversal learning in head-fixed mice within the MHC

**Figure 4:**
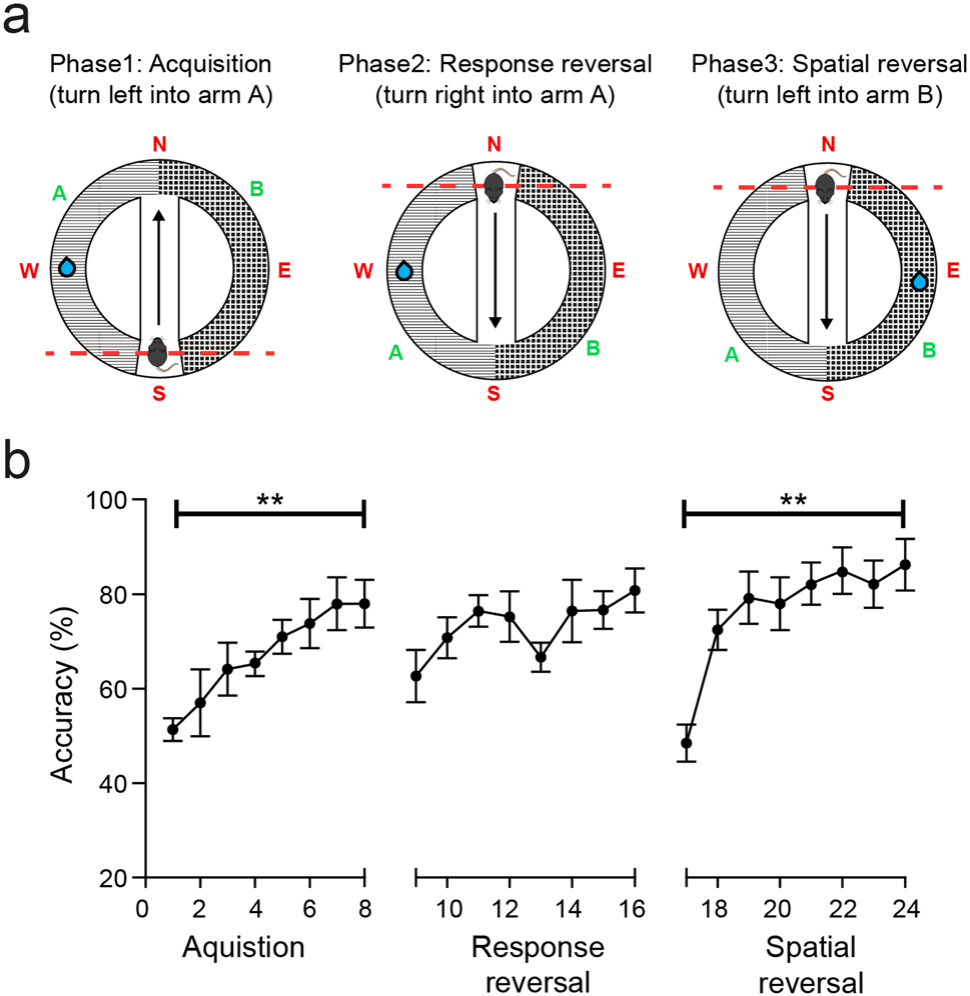
Head-fixed mice show flexible spatial reversal learning in MHC in the absence of external maze cues. (**a**) Schematic of the T maze during each phase of learning, depicting an example starting point (mouse), reward delivery location (blue droplet), and the direction of head-fixation oriented toward the four cardinal points [North (N), East (E), South (S), West (W)]. The dotted red line represents the head-fixation bridge. Note the position of the doors separating the choice arms (**a**,**b**) and the starting point can be manipulated such that the left/right orientation of the choice arms can be switched relative to the direction of approach through the central corridor (arrows). (**b**) Significant spatial learning occurred across the 8 acquisition sessions in phase 1. In phase 2, rotating the head-fixation by 180 such that the animals were required to make the reverse egocentric response to obtain reward from the same choice arm, resulted in an initial drop in accuracy that increased over sessions and reached the same level as in phase 1. In phase 3 the location of reward delivery was changed such that the animals were required to make the reverse egocentric response again to obtain reward from the previously unrewarded arm. Animals showed a larger reduction in accuracy that again resolved over sessions. Data shown as mean ± SEM, n = 6. **p < 0.01.

To further test spatial navigation in the MHC we carried out a simple two-choice response discrimination task using a T-maze style track, where the animals were rewarded for turning into one of two choice arms (Experiment 1, Fig. 4a). The mice generally showed improvement in accuracy of discrimination learning during the acquisition phase over the 8 sessions (F7,35 = 6.2, p = 0.008), however one animal was excluded after being unable to reach more than 66% accuracy (8/10 correct trials).

We next assessed the flexibility of the animals’ egocentric responses by altering the starting position of the mice within the procedure room (by 180° rotation of the head fixation bridge) and relative to the choice arms (by moving the position of the T maze doors). The animals were thus required to perform the opposite egocentric response to obtain reward from the same choice arm as in the acquisition phase (response reversal). We found that while there was a drop in accuracy compared to the previous acquisition day, overall the animals tended to perform above chance (Fig. 4b, t5 = 2.28, p = 0.072), indicating that they were not relying solely on a previously learned egocentric response to make accurate choices. Accuracy tended to improve again across the subsequent sessions, however this did not reach statistical significance (most likely driven by the higher baseline accuracy on the first response reversal day).

The animals then underwent a spatial reversal whereby the location of the reward was moved to the previously unrewarded arm. In this instance the animals were required to learn new place information as well as reverse their egocentric response. This resulted in large reduction in accuracy on the first day of this phase followed by significant improvement in accuracy over time (Fig. 4b, F7,35 = 14.7, p < 0.0001). Overall, the data suggest that mice are capable of flexible spatial learning in the MHC despite a lack of the reliable extra-maze information that would be afforded in freely moving discrimination tasks.

### Dependence of spatial reversal learning on hippocampal acetylcholine

**Figure 5:**
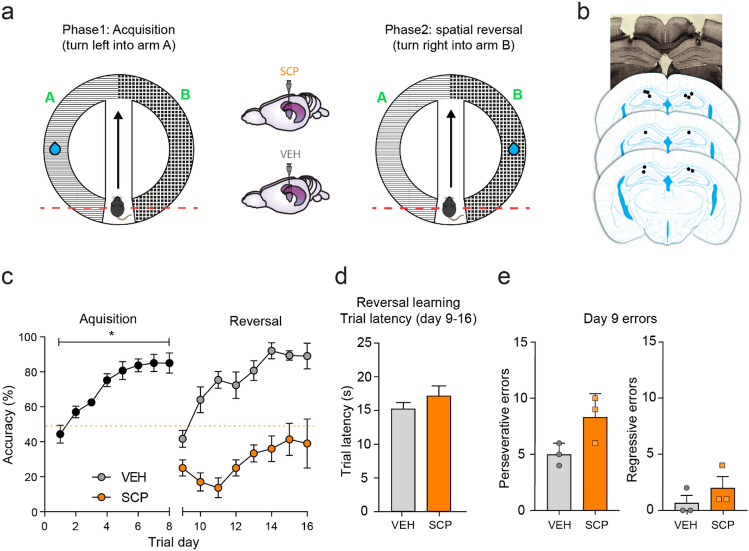
Disruption of hippocampal acetylcholine impairs spatial reversal learning in the MHC. (**a**) Schematic of the T maze during acquisition and spatial reversal learning, depicting an example starting point (mouse) and reward delivery location (blue droplet). The dotted red line represents the head-fixation bridge. Note the position of the doors separating the choice arms (**a**,**b**) and the starting point was manipulated such that the left/right orientation of the choice arms was switched relative to the direction of approach through the central corridor (arrows). 5 min prior to phase 2 sessions, animals received a bilateral infusion of scopolamine (1 µM) or vehicle (PBS) into the dorsal hippocampus. (**b**) Location of the injector placement within the hippocampus was confirmed post-mortem (*top*: representative photomicrograph, *bottom*: black dots show locations from all animals mapped onto coronal sections). (**c**) Spatial learning occurred in all 6 animals (then split into counterbalanced groups). During phase 2, scopolamine-treated animals were unable to acquire the new reward location as evidenced by decreased accuracy. (**d**) There is no evidence to suggest that scopolamine increased the overall trial latency compared to control treatment (data pooled across 8 training session within each learning phase). (**e**) Analysis of error types during reversal learning showed that scopolamine treatment increased perseverative errors compared to the control group. Data shown as mean ± SEM, *p < 0.05 RM ANOVA, n = 3/treatment group.

We next investigated the effect of disrupting hippocampal cholinergic signalling in the MHC spatial reversal learning task using the non-selective muscarinic antagonist, scopolamine. We showed that the animals once again readily acquired the discrimination learning task (Experiment 2, Fig. 5a) as evidenced by an increase in accuracy over the 8 days of acquisition training (F_7,35_ = 21.0, p < 0.0001; Fig. 5c). During the second learning phase animals underwent spatial reversal learning by relocation of the reward to the opposite choice arm (Fig. 5a). Infusion of scopolamine into the dorsal hippocampus (Fig. 5b) prior to each reversal learning session resulted in significant impairment in relearning of the new reward location compared to animals receiving vehicle infusion (Fig. 5c). This was reflected by significant main effects of DRUG (F_1,4_ = 55.1, p = 0.002) and DRUG x DAY interactions (F_7,28_ = 2.95, p = 0.019) in choice accuracy. There was no effect of scopolamine on trial latencies during the reversal learning phase (Fig. 5d). Further analysis of the type of errors made during the initial reversal learning session (D9), showed that scopolamine-treated animals tended to show perseverative responses on the previously rewarded choice arm compared to the vehicle-treated group, however this did not reach statistical significance (t_4_ = 2.5, p = 0.067). There was no effect of treatment on regressive errors (Fig. 5e).

## Discussion

In this study we show that mice are able to navigate in 2 dimensions within an air-lifted floating real-world environment. Head-fixed mice were able to represent their spatial location without use of distal visual cues as demonstrated by place cell recordings made from CA1 neurons in the hippocampus. They were then able to use these hippocampal representations to perform a reversal learning spatial navigation task. Thus, we demonstrate that the MHC environment allows performance of complex spatial navigation tasks in head-fixed mice whilst providing the physical stability for whole-cell patch clamp recordings lasting 10 s of minutes.

Head-fixed recordings allow high resolution 2-photon imaging and electrophysiological recordings of neuronal activity during behavior. However, head fixation necessarily places constraints on the types of behavior that can be performed. Many groups have developed head-fixed behavioral tasks where the body of the mouse is supported and essentially immobile and where cognitive processes are assessed in response to visual or auditory cues by manipulating levers or wheels with the front paws, or direction of licking (e.g.^34–38^). Such methods can deliver highly reproducible complex behaviors but are not so useful for testing spatial navigation which is uniquely amenable to testing in rodents. Because of the attractiveness of the hippocampal place cell spatial navigation system as a model to probe the neural mechanisms of cognition, many groups have sought to combine head fixation with spatial navigation tasks. This marriage has been achieved with the use of virtual reality systems where mice run on a treadmill, fixed wheel or floating ball whose movement dictates the flow of visual cues and/or other sensory cues such as olfactory or somatosensory cues^1,3,4,13,29,39^. Virtual reality systems are shown to work well if mice are restricted to moving on a linear track in 1 dimension but mice perform much less well if the virtual arena expands to an open field of 2 dimensions^7,19^. One way around this problem is to create virtual linear tracks with branch points (analogous to real world T-maze configurations) where mice are required to choose an arm of the maze based on prior experience^20^. Alternatively, the underlying problem for navigating in virtual reality with head position fixed is proposed to be the lack of vestibular head direction sensory input^7^. This can be resolved by using a swivel mount on the head clamp allowing the head direction to rotate, which enables rodents to navigate successfully in 2 dimensions^9,10,19^. In the MHC, mice successfully navigate in 2 dimensions without rotation in the head direction implying that using a real-world physical environment with associated somatosensory cues removes the need for head rotation and vestibular head direction movement.

Head-fixation is an unnatural configuration for rodents that might engender enhanced stress responses interfering with behavioral experiments. There is some evidence that stress is increased but the majority of observed behavioral and hormonal stress response in the air lifted MHC may be ameliorated by appropriate habituation and training^8^. In this study we have reduced stress levels further by introducing an angled head clamp that produces a natural head posture in mice where the whiskers are able to engage with both the ground and walls of the arena. This provides the natural somatosensory input that mice rely heavily on for navigation. We have also introduced a vizor above the eyes to exclude visual cues from overhead that performs the dual function of preventing access to distal stationary cues external to the MHC and reducing overhead visual movement cues that typically elicit a fear response. Furthermore, the posture of mice within the MHC is relatively stable and natural with all 4 limbs engaged with the flat surface. These adaptations, combined with the observed reduction in stress levels with habituation^8^, indicate that the air lifted MHC provides the least stressful head-fixed recording configuration for rodents.

The place cell data in this study indicates that mice experience the MHC arena and form hippocampal representations in a similar way to freely moving exploration. This is further supported by measurement of place cell activity in the MHC using 2-photon calcium imaging^24^. The imaging data reveal stable place cell representations over days and place cell remapping in novel environments. Moreover, the spatial information content of place cell activity is similar to that found in freely moving rodents^40–42^ as confirmed by our data.

The MHC arena also provides a physically stable platform in which to perform whole-cell patch clamp recordings from head-fixed navigating mice. We show that recordings may routinely be maintained for 10–20 min, sufficient to gain insight into the sub-threshold and supra-threshold membrane potential dynamics of CA1 pyramidal cells. This has the potential to measure excitatory and inhibitory synaptic inputs during spatial learning paradigms^1,41,43^ and the occurrence of plateau potentials thought to be critical for driving plasticity of place cell representations^29,30^.

Reversal learning in the rewarded T-maze configuration of the MHC requires the remapping of reward location representations. We show this is hippocampal-dependent and specifically that cholinergic signalling in the hippocampus is required. Acetylcholine release and cholinergic receptor activation has been shown to be important for memory formation and synaptic plasticity in the hippocampus^44,45^. In this model, acetylcholine released in response to surprise or uncertainty^38,46,47^ signals the need to learn new associations and achieves this by facilitating synaptic plasticity. The mechanism of facilitation is multifactorial with acetylcholine reorganising interneuron networks^48^, altering the relative balance of input pathways^48,49^, enhancing cellular and dendritic excitability^50,51^ and facilitating NMDA receptor function and signalling leading to the expression of plasticity^33,52^. In the context of our experiments, performance in the reversal learning task requires remapping of reward location that depends on synaptic plasticity facilitated by acetylcholine release in the hippocampus.

## Methods

### Animals

All procedures were performed in accordance with the UK Animals Scientific Procedures Act (1986) and were approved by the University of Bristol Animal Welfare and Ethical Review Board. All animal experiments are reported according to the ARRIVE guidelines. Adult, male C57BL6/J mice were purchased from Charles River, UK. Following at least 1 week of habituation to the University of Bristol facility, surgery was performed between 8 and 10 weeks of age. All mice were housed on a 12-h light cycle (lights off at 8:00am), initially, 2 per cage. Immediately after the head-plate/cannulation/craniotomy surgery, mice were separated and housed individually for 2 days during recovery, then returned to their previous pair-housing for the remainder of the study. Access to rodent chow and water was provided ad libitum unless undergoing the water restriction procedure outlined below. All behavioral experiments were performed under red illumination during the animal’s dark phase, between 10:00 and 12.00 h. All animals were sacrificed at the end of experiments by lethal dose of anaesthetic (isoflurane).

### Drugs

Scopolamine hydrobromide (Sigma Aldrich, UK) was dissolved in sterile 0.1 M PBS to 1 μM and administered bilaterally into the dorsal hippocampus at a volume of 0.5 μl per side, 5 min prior to behavioural testing. Control animals received an infusion of PBS alone. Treatments were randomised across mice and the experimenter was blind to treatment condition.

### Stereotaxic surgery

Surgery to implant the head-plate was performed a week before the beginning of head-fixed experiments. For experiments involving drug infusion into the dorsal hippocampus, mice were also implanted with an infusion cannula during the same surgery. Animals were anaesthetised with isoflurane and secured in a stereotaxic frame. The skull surface was exposed by removing a small piece of skin between bregma and lambda, and cleaned with 3% Hydrogen peroxide. The edges of the skin were secured to the skull surface using Vetbond. The skull surface was lightly scored with a scalpel and a headplate (model9, Neurotar, Finland) secured to the skull with superglue. A 1.5 mm diameter craniotomy was drilled over the dorsal hippocampus and for electrophysiology experiments plugged with agar. In animals used for the infusion study, a bilateral 32-gauge guide cannula (Plastics One, UK) was then implanted in the dorsal hippocampus according to the stereotaxic coordinates: AP ML and DV from bregma (Paxinos and Watson, 1998). The resulting placement of the infusion needle extended 0.5 mm below the cannula. The headplate/cannula were then secured to the skull with dental acrylic. After surgery the animals were housed individually for 48 h then allowed a further 3–5 days recovery in normal pair housing conditions.

### Head-fixation

Five days after surgery, mice were handled for 15-min sessions per day. They were removed from their home cage using a tunnel (the familiar cardboard tube present in all cages) and held with the experimenters’ palms at either end. The mouse was allowed to explore the openings of the tunnel and if they left, the tunnel was removed so that they could explore the experimenter’s cupped hand. The animal was then placed in the carbon-fibre arena used in the MHC and allowed to freely explore for 5 min.

Once the mice were observed to readily return from the MHC arena to the experimenter’s hand, they then underwent habituation to the transfer cloth on the following day. All mice reached this stage by the third session of handling. The mice were habituated to the cloth used for transfer to the head-fixation by placing them on the cloth and wrapping and unwrapping several times (< 20 s each) before being returned to their home cage. Head-fixation commenced the following day with mice wrapped in the cloth and positioned in the head-fixed apparatus. The head-fixed apparatus was the Mobile HomeCage (MHC) system (Neurotar Ltd, Finland) where animals are head-fixed to an aluminium frame over an ultralight, air lifted carbon fiber container (diameter 34 cm). The track shape (circular or T-maze) was changed using custom foam inserts and tracks were decorated with visual cues (stripes and spots) to enable visual discrimination of location. Once the mouse was positioned under the aluminum frame, the head-plate was clamped in the head-post, the floating container was placed underneath, the cloth wrapping was removed, and the air-compressor was turned on. The air-compressor generated noise of around 50 dB, and the pressure was adjusted to allow a sheet of paper to just fit between the base of the air table and the carbon-fibre arena. In all experiments the head-fixed sessions were performed on consecutive days and lasted for up to 20 min.

### Water restriction

In order to motivate the mice to perform reward-driven trials in the MHC, they underwent a water restriction protocol commencing the day prior to the first head-fixation session, and lasting for the duration of the study. Water was removed from the homecage ~ 16 h prior to head-fixation in the MHC the following day, and was returned at least 1 h after the animal had been returned to their homecage following head-fixation. The animals had free access to water for ~ 4 h before it was removed again in advance of the following day’s training/testing. Body weights were recorded at two time points: immediately prior to head-fixation, and after the 4 h of ad libitum water.

### MHC training

All experimental animals began training in the MHC using a circular foam insert that represented a continuous linear track. On day 1 the mice were head-fixed and allowed to move the air-lifted container for 15 min or until their movement was observed to stabilise (e.g. no struggling against the head-clamp, no backward movement or spinning of the container). No reward was provided in this session. On day 2 mice were head-fixed for 15 min and a sucrose reward (~ 3 μl of a 10% solution) was delivered through a lick port positioned at the left side of the mouth each time the animal made a complete traversal of the track. Since the animals usually moved the container in only one direction in this session (e.g. clockwise), and rarely turned within the track, on day 3 the container was placed beneath the mouse such that the animal was required to move it in the opposite direction (e.g. anticlockwise) to traverse the track. The purpose of this was to train the animal to move the container in both directions, to reduce the likelihood of spatial bias occurring during T maze trials.

All mice were then habituated to the T maze container before commencing experimental acquisition trials. The T maze container consisted of the circular track with an additional central corridor that was accessed at one end via one way doors and was open to the circular track at the other end. Mice were head-fixed in the MHC and allowed to freely explore the maze. Passing through the reward zone on either arm resulted in the delivery of reward through the lick port. On arriving at the end of the choice arm the mice were manually assisted in passing through the door by the experimenter by gently moving the maze under the mouse until they pushed open the door. On succeeding trials, manual assistance was applied only if the mouse failed to pass through the door for 1 min. All mice were able to pass through the door unaided by the end of the habituation session. The mouse was removed from head-fixation once both arms had been visited on at least 5 trials each. In order to reduce the likelihood of spatial bias developing during the experimental acquisition phase, the choice arm that had been visited by the mouse on fewer trials during the habituation session was allocated as the rewarded arm during acquisition trials.

### Electrophysiology

#### Extracellular place cell recording and analysis

Following head fixation, the cortical surface was exposed by removal of the agar plug from the craniotomy and a 4-shank, 64-channel silicon probe (chronic H-2 probe, Cambridge NeuroTech, UK) centered on − 2 mm, + 1.25 mm from bregma. The probe was connected to an Open Ephys acquisition board via an RHD 64ch headstage (Intan Technologies). The probe was lowered at approximately 20 µm/min until prominent theta and/or sharpwave-ripple activity was observed in LFP. The probe was then adjusted at 2–5 µm per minute to optimise the number and amplitude of extracellular action potentials on deeper recording channels and left in position to stabilize for at least 15 min before recording began. After recording, the mouse was deeply anesthetized with sodium pentobarbital and + 30 mA current used to mark recording sites with electrolytic lesions, identified in histological sections following transcardial fixation perfusion.

Extracellular action potentials were isolated using Kilosort2 (https://github.com/jamesjun/Kilosort2) followed by manual refinement using Phy (https://phy.readthedocs.io/en/latest/); Isolation Distance and L-ratio were used to validate isolation. The CircStats.m toolbox was used to calculate phase of spiking relative to 5-8 Hz theta oscillations derived from an LFP channel in the CA1 pyramidal cell layer. Spikes were assigned to positions on the annular track by synching video and Open Ephys data streams using a TTL/LED pulse, and spatial information content calculated according to Eq. 1 (below,^26^).

Estimation of mouse location using place cell population activity was based directly on the Bayesian decoding framework detailed in^53^, hence founded on the standard Bayes rule of conditional probability:

$$ P({\mathbf{x}}|{\mathbf{n}})P({\mathbf{n}}) = P({\mathbf{n}}|{\mathbf{x}})P({\mathbf{x}}) $$

The objective of reconstruction is to calculate P(**x**|**n**), the probability of the animal being at position **x**, given the spikes fired during any given time bin. P(**x**) is the probability the animal occupies that locational bin at that time and is calculated experimentally through spatial occupancy. P(**n**) refers to the probability of n number of spikes occurring. For positional binning, the annular track was subdivided into 30 positional bins, each occupying 12 track degrees. Following^53^, the firing rates of individual cells were assumed to be independent of one other and follow a Poisson distribution. Under these assumptions, P(**n**|**x**) is given by:

$$ P({\mathbf{x}}|{\mathbf{n}}) = \prod\limits_{i = 1}^{N} {P(n_{i} |{\mathbf{x}})} = \prod\limits_{i = 1}^{N} {\frac{{(\tau f_{i} ({\mathbf{x}}))^{{n_{i} }} }}{{n_{i} !}}} \exp ( - \tau f_{i} (x)) $$

Here, f_i_(x) is the average firing rate of the neuron i at position x, and τ is the duration of the time window (500 ms for our analyses). Using this above expression to calculate the final probability gives:

$$ P({\mathbf{x}}|{\mathbf{n}}) = C(\tau ,{\mathbf{n}})P({\mathbf{x}})\left( {\prod\limits_{i = 1}^{N} {f_{i} ({\mathbf{x}})^{{n_{i} }} } } \right)\exp \left( { - \tau \sum\limits_{i = 1}^{N} {f_{i} ({\mathbf{x}})} } \right) $$

The method produces a probability distribution of position in each time bin, τ; the most probable position is chosen as the reconstructed location. The time bin stepped forward by 250 ms to decode the next position in the reconstructed trajectory, ignoring times when the mouse was stationary to exclude potential non-local representations during sharp wave ripples.

#### Whole-cell patch clamp recording and analysis

Whole cell voltage clamp experiments were conducted in head-fixed mice. The cortical surface was exposed via removal of an agar plug above a craniotomy and recording chamber previously created during headplate fixation. Craniotomies were filled with aCSF and a silver chloride ground electrode placed within the recording chamber. Glass recording electrodes with a resistance of 6–7 MΩ were pulled from borosilicate glass capillaries filled with internal solution were slowly lowered into the brain with a positive electrode pressure of 300 milibar. Electrodes were lowered to a depth of approximately − 1.2 mm DV after which positive pressure was reduced to 0.02 milibar. Recording electrodes were then slowly lowered in 2 μm steps until the pipette resistance decreased on three consecutive steps, upon which Gigaseal formation and whole cell access was achieved as with standard patch clamp techniques. Neurons were recorded in current clamp mode using a Axoclamp 700A amplifier (molecular devices) and digitised at a sampling frequency of 25 kHz with a micro 1401 data acquisition board (CED). Data was acquired using spike2 software (CED; https://ced.co.uk/products/spkovin) and analysed via custom MATLAB scripts. For place cell induction in vivo a 750 ms current step was injected into the neuron whilst the mouse was running within the MHC. Spike times and MHC tracker data were aligned and velocity data filtered to running and stationary epochs when mouse velocity was above or below 25 cm/s. Membrane potential measurements were acquired by removal of action potentials and linear interpolation across a 3.5 ms window starting 1 ms before action potential initiation. Burst and complex spikes were classed as continuous spikes with an inter-spike interval less than 100 ms and ramp depolarizations were calculated and the membrane voltage integral 100 ms before the onset of the detected spike set.

For place cell analysis spike timings were grouped into 30 positional bins of 12 track degrees and mean firing rate during running epochs calculated as a gaussian smoothed firing rate and occupancy of the mouse in each bin.

Spatial information (Eq. 1) for the recorded neuron was calculated for the whole recording, the first and second half of the recording.

1 $$ Spatial \, information = \mathop \sum \limits_{i} p_{i} (r_{i} /\overline{r}) \cdot {\text{log}}_{{2}} (r_{i} /\overline{r}) $$

where p_i_ and r_i_ is probability and firing rate of the mouse/cell in position bin i and $\overline{r }$ the mean firing rate.

Spatial information values were compared to a distribution of spatial information acquired by shuffling spike times 500 times. Statistically significant spatial coding was calculated using a Z-test comparing the recorded spatial information to the randomly shuffled spatial information population.

Underlying membrane potential and theta power from running epochs were binned across spatial bins. Theta power was calculated via multi taper Fourier transform and taken as the average power between 5 and 8 Hz.

### Experiment 1

#### Acquisition

Animals were head-fixed in the T maze container at the starting point and held in place by the experimenter until recording started (< 30 s). The animal was then allowed to proceed along the central corridor and travel down 1 of the 2 choice arms. If the ‘correct’ arm was chosen, the mouse received a droplet of 10% sucrose (~ 3 μl) delivered via the lick port. The animal then passed through the door at the end of the choice arm and the trial was re-started. No reward was received in the opposite ‘incorrect’ arm, or if the mouse passed back through the choice arm to the reward location. Each mouse completed 12 trials and was removed from head-fixation after the passing through the door on the 12th trial. The acquisition phase comprised 8 sessions of 12 choice trials. Accuracy was calculated as the percentage correct trials in each session.

#### Response reversal

During the response reversal phase the head-fixation bridge was rotated by 180 degrees such that the head direction of the mouse was fixed in the opposite direction to the acquisition phase. The doors on the T maze were moved to the opposite end of the central corridor so that the left/right configuration of the cued choice arms was reversed from the perspective of the mouse approaching the choice point. The designated ‘correct’ arm remained the same as in acquisition trials, therefore the animals were now required to make the opposite egocentric response to obtain reward from the previously learned arm. The response reversal trials were otherwise carried out as previously, with 12 trials per day across 8 daily sessions.

#### Spatial reversal

During the spatial reversal phase, the head-fixation bridge and T maze doors were maintained in the same position as in the previous response reversal phase, however the designated ‘correct’ arm was switched, therefore the animals were required to make the opposite egocentric response again in order to receive reward in the previously un-rewarded arm. Another 8 sessions of 12 trials/day were carried out in this configuration.

### Experiment 2

#### Acquisition

All animals underwent the same acquisition trials as in experiment 1. At the end of the learning phase the animals were split into two groups counterbalanced for learning ability.

#### Spatial reversal

Animals were head-fixed in the MHC and received a pre-treatment infusion of either scopolamine or vehicle prior to spatial reversal trials. The circular track container was used so that the animals were able to move around the environment during the infusion procedure, and were only placed in the T maze when the reversal trials commenced. The position of neither the head-fixation bridge nor the doors was changed relative to the acquisition phase, however the designated ‘correct’ arm was switched such that the animals were required to make the opposite egocentric response into the previously unrewarded arm to obtain reward. The animal received 12 trials/day for 8 sessions, and the experimenter was blind to drug treatment throughout. Trial outcomes were recorded by the experimenter and later confirmed from tracker recordings obtained using. Trial latencies were extracted from the tracking data using a custom script in Matlab. Perseverative and regressive error types were analysed to determine whether the treatment altered perseveration or reversions back to the previously correct choice arm. This analysis has previously been used in studies where a single reversal session is applied, therefore error types were determined from trials on the first reversal session (D9) only. Perseveration involved continuing to make the same egocentric response as required during the acquisition phase and was defined as entering the incorrect arm in 3 or more trials in consecutive blocks of 4 trials. Once the mouse made less than 3 errors in a block for the first time, all subsequent errors were counted as regressive errors. This allowed a measure of the ability to maintain a new choice after initially shifting away from the previously learned choice.

### Infusion procedure

Prior to training in the T maze, mice were habituated to the infusion procedure during the MHC training sessions on the circular training track. At the beginning of each session, once the animals were head-fixed, the cannula dummy was removed and a 33-gauge bilateral injector extending 0.5 mm beyond the length of the guide cannula was inserted into the dorsal hippocampus and left for 1 min before being removed and the dummy replaced. On the last session prior to acquisition training in the T maze, the animals also received a vehicle infusion. The injector was inserted and left in place for 1 min prior to infusion of PBS (0.5 μl over 1 min). The injector was left in place for a further 1 min to allow diffusion of the drug into the surrounding tissue, and then the injector was removed and the dummy replaced. During experimental infusions either vehicle or scopolamine (1 μM) was administered in the same manner 5 min before the animal was transferred from the circular track to the T maze to begin choice trials.

### Histology

Following completion of the experiment, all mice received an infusion of black India ink via the implanted cannula to enable the final location of the infusion to be visualised. Animals were anesthetised with a lethal dose of sodium pentobarbitone (0.1 ml Euthatal, 200 mg/ml) and perfused via the left ventricle with 0.01 M PBS followed by 4% paraformaldehyde. The brains were removed and postfixed in 4% paraformaldehyde overnight at 4 °C. Prior to being cut, the brains were transferred to 30% sucrose in 0.1 M PBS and left for up to 48 h until they had sunk. Coronal sections (50 μm) were cut on a freezing microtome. Locations of the final injector tip positions in the hippocampus were determined under a light microscope mapped onto standardised coronal sections of a mouse brain stereotaxic atlas (Paxinos and Watson, 1998).

### Statistical analysis

Data in the text and in the figures are presented as mean ± SEM and the level of significance was *p* < 0.05 for statistical comparisons of all datasets. In experiment 1, when one factor was measured at different times for a single group, one-way RM ANOVA followed by Bonferroni’s post hoc tests was used. The behavioural performance of each learning phase was separately analysed. In Experiment 2 a two-way RM ANOVA (within factor: session, between factor: treatment) followed by Bonferroni post hoc tests for multiple comparisons were used to assess the effects of scopolamine on correct arm choices in the reversal learning phase. Trial latencies were pooled across the reversal learning sessions and subjected to unpaired t-test to compare the effect of drug treatment. Error types were analysed by unpaired t-test from the first reversal learning session (D9). Data analysis was performed with GraphPad Prism 8.4.2 (GraphPad Software, CA, US).

## Data Availability

The datasets generated and analysed during the current study will be published alongside the manuscript and are freely available from the corresponding author on request.
